# Supplementary material for: Identification of ER:Melanosome Membrane Contact Sites in the Retinal Pigment Epithelium
Source: Contact (Thousand Oaks). 2025 Jun 2;8:25152564251340949. doi: 10.1177/25152564251340949 (PMC12130655; doi:10.1177/25152564251340949)
Supplement: sj-pptx-1-ctc-10.1177_25152564251340949 - Supplemental material for Identification of ER:Melanosome Membrane Contact Sites in the Retinal Pigment Epithelium [file sj-pptx-1-ctc-10.1177_25152564251340949.pptx]

## Slide 1
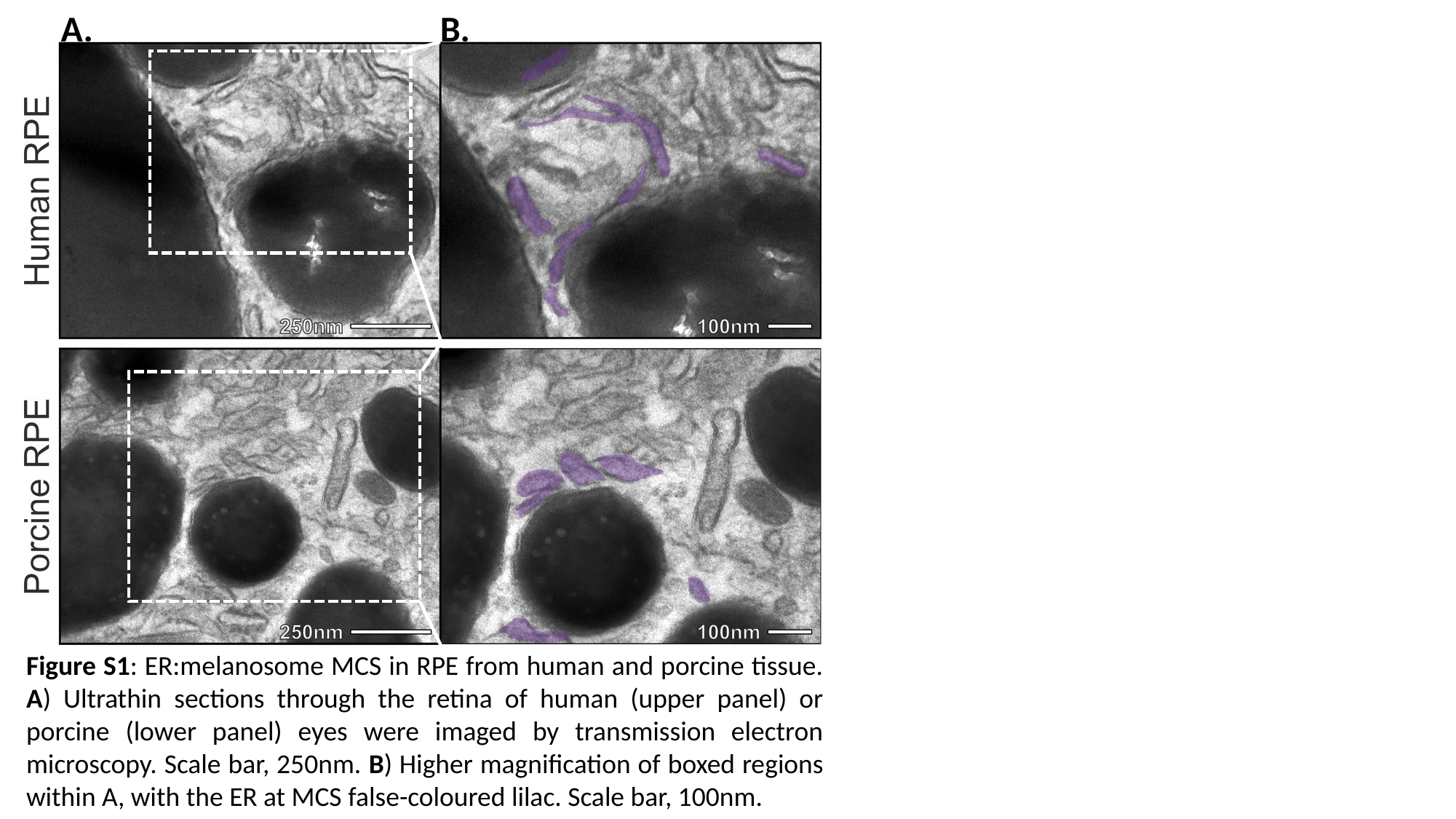

A.
B.
Figure S1: ER:melanosome MCS in RPE from human and porcine tissue. A) Ultrathin sections through the retina of human (upper panel) or porcine (lower panel) eyes were imaged by transmission electron microscopy. Scale bar, 250nm. B) Higher magnification of boxed regions within A, with the ER at MCS false-coloured lilac. Scale bar, 100nm.
