# Supplementary material for: Identification of ER:Melanosome Membrane Contact Sites in the Retinal Pigment Epithelium
Source: Contact (Thousand Oaks). 2025 Jun 2;8:25152564251340949. doi: 10.1177/25152564251340949 (PMC12130655; doi:10.1177/25152564251340949)
Supplement: sj-pptx-2-ctc-10.1177_25152564251340949 - Supplemental material for Identification of ER:Melanosome Membrane Contact Sites in the Retinal Pigment Epithelium [file sj-pptx-2-ctc-10.1177_25152564251340949.pptx]

## Slide 1
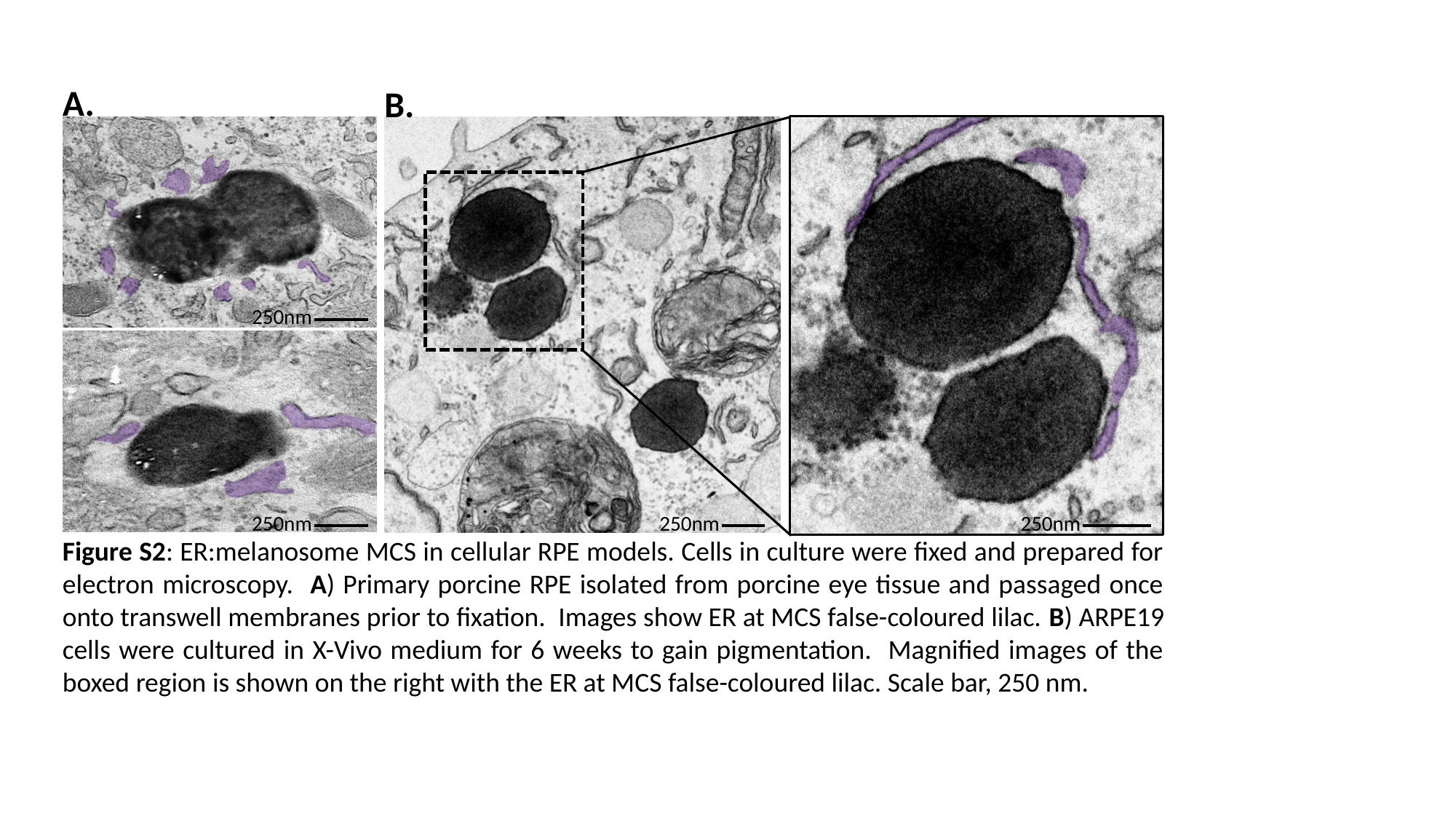

A.
B.
250nm
250nm
250nm
250nm
Figure S2: ER:melanosome MCS in cellular RPE models. Cells in culture were fixed and prepared for electron microscopy. A) Primary porcine RPE isolated from porcine eye tissue and passaged once onto transwell membranes prior to fixation. Images show ER at MCS false-coloured lilac. B) ARPE19 cells were cultured in X-Vivo medium for 6 weeks to gain pigmentation. Magnified images of the boxed region is shown on the right with the ER at MCS false-coloured lilac. Scale bar, 250 nm.
